# Impact of COVID-19 Pandemic on Emergency Department Visits for Opioid Use Disorder Across University of California Health Centers

**DOI:** 10.5811/westjem.18468

**Published:** 2024-10-02

**Authors:** Matthew Heshmatipour, Ding Quan Ng, Emily Yi-Wen Truong, Jianwei Zheng, Alexandre Chan, Yun Wang

**Affiliations:** * University of California Irvine, School of Pharmacy and Pharmaceutical Sciences, Irvine, California; † University of California Irvine, Donald Bren School of Information and Computer Sciences, Irvine, California; ‡ Chapman University, School of Pharmacy, Irvine, California

## Abstract

**Introduction:**

Coronavirus 2019 (COVID-19) has had a devastating impact on mental health and access to addiction treatment in the United States, including in California, which resulted in the highest rates of emergency department visits (ED) for opioid poisoning in 2020. As California slowly returns to pre-pandemic normalcy, it remains uncertain whether the rates of opioid-related events have slowed down over time. We hypothesized that the number of opioid-related ED visits were exacerbated after the period of the COVID-19 pandemic and continue at a high rate in the present.

**Methods:**

In this analysis we searched the University of California (UC) Health Data Warehouse—a database of electronic health records from six academic medical centers—for opioid related ED visits, identifiying using the following International Classification of Diseases, 10^th^ Ed, Clinical Modification codes: F11 codes, and T40.0*, T40.1*, T40.2*, T40.3*, T40.4*, T40.6*. Opioid overdose-associated visits were classified by types of opioids involved: heroin (T40.1*); prescription opioids (T40.2* or T40.3*); and synthetic opioids (T40.4*). We performed interrupted time analysis to estimate the immediate (level) change and change-in-time trend (trend change), from before (January 2018–October 2019) and during the pandemic (April 2020–December 2022). Monthly visit rates were evaluated with negative binomial regression adjusted for first-order autoregression and using all-cause ED counts as the offset. We present effect sizes as rate ratios (RR) and 95% confidence intervals (CI), tested at α = .05.

**Results:**

We observed a decrease in overall ED visits from 28,426 to 25,121 visits in December 2019 and June 2021, respectively across all six UC Health Centers. Before COVID-19, we found that ED visit rates steadily increased for all outcomes (*P* < 0.05) except synthetic opioids. Total opioid-related ED visit rates increased by 15% (RR 1.15, 95% CI 1.02–1.29, *P* = 0.20) immediately after March 2020 before decreasing by 0.5% every month, albeit without statistical significance (RR .995, 95% CI .991–1.00, *P* = 0.06). Opioid-related events across the six academic medical centers increase from 232 in December 2019, representing a single month’s total, and peaked at 315 in June 2021. Similar trends were observed with prescription opioid overdoses, with a step increase of 44% (RR 1.44, 95% CI 1.10–1.89, *P* = .008) before plateauing after March 2020 (RR 1.01, 95% CI .998–1.02, *P* = 0.12). Specifically, the total number of prescription opioid-related ED visits more than doubled between December 2019 (22 visits) and June 2021 (49 visits). After March 2020, ED visit rates for synthetic opioid overdoses were increasing steadily by 4% every month (RR 1.04, 95% CI 1.02–1.06, *P* = .001), unlike with heroin, which was observed with an 8% monthly reduction (RR .92, 95% CI .90–.93, *P* < .001). No immediate increase in visit rates was observed for either opioid.

**Conclusion:**

While opioid-related ED admissions among the UC health centers showed an overall decrease, prescription and synthetic opioid overdoses remained significantly higher than pre-pandemic trends as of December 2022. A multilevel approach to improve awareness of new opioid health policies could ameliorate these alarming rises in the post-pandemic era.

Population Health Research Capsule What do we already know about this issue?

*The opioid use disorder (OUD) epidemic and COVID-19 pandemic are two public health crises that significantly increased ED visits in the US.*

What was the research question?

*How did the COVID-19 pandemic affect opioid-related ED visits in California?*

What was the major finding of the study?

*Opioid prescriptions and heroin-related ED visits increased after the pandemic across UC hospitals.*

How does this improve population health?

*Our findings show trends of opioid use in California and identify key community elements that could contribute to OUD interventions through future research.*

## INTRODUCTION

Opioids play a major role in healthcare as an important prescription medication for pain relief. Opioid analgesics are a beneficial intervention when properly administered,^1^ thus creating challenges when regulating their use as they have the potential for long-term adverse effects. Although earlier phases of the opioid crisis were characterized by the misuse of prescription opioids (first wave), recent trends reveal that heroin (second wave) and illicit synthetic opioids (third wave) have become crucial to characterizing the opioid epidemic.^2^ Synthetic opioids such as fentanyl have been shown to be the most devastating contributors to the current rising opioid-related cases due to its associated positive supply shock, allowing for its price to reduce significantly.^3^ Predicted trends overlaying scatterplots.

Beginning in early 2020, the advent of the coronavirus 2019 (COVID-19) pandemic led to an abrupt, worldwide disruption to societal functions and typical daily life as stay-at-home orders were implemented to curb the spread of the virus and preserve medical facilities and equipment for the most severe infections. The subsequent rise in unemployment rates and social isolation led to increased psychological distress,^4^ which was postulated to have caused a nationwide increase in opioid overdose cases across multiple states in 2020.^5^ ^–^ ^8^ As society slowly began to return to pre-pandemic normalcy in 2021 and 2022, it remains uncertain whether the rates of opioid-related events have slowed over time, given the challenges of weaning off chronic opioid use. Thus, a deeper exploration into the trends related to opioid-related events in 2021 and 2022 and a review of current interventions and solutions is necessary to allocate resources for enhancing our management of the opioid epidemic.

In this paper we report our findings for emergency department (ED) visit rates associated with opioid-related cases from 2018–2022 across the six University of California (UC) health centers. We compared the rates from the period before the pandemic (January 2018–December 2019) with those during the pandemic (April 2020–December 2022). A washout period between January–March 2020 was implemented due to widespread public uncertainty regarding the nature of the pandemic during that timeframe. These dates take reference from the US’s first confirmed laboratory case on January 20, 2020, and California’s statewide stay-at-home order on March 19, 2020.^9^ We hypothesized that opioid-related ED visit rates continued to worsen after 2021 and remained higher than pre-pandemic rates as of December 2022.

## MATERIALS AND METHODS

### Data Source

For this analysis we used the UC Health Data Warehouse (UCHDW), a database of electronic health records from the six UC academic health centers: UC Davis; UC Irvine; UC Los Angeles; UC Riverside; UC San Diego; and UC San Francisco. The UCHDW contains clinical data of 8.7 million patients seen at a UC facility, totaling approximately 378 million encounters including office visits, inpatient admissions, and ED visits.^10^ All data is organized based on the Observational Medical Outcomes Partnership (OMOP) common data model (CDM), an open community data standard for standardizing the structure of real-world clinical data across institutions despite differences in the underlying clinical data infrastructure.^11^ Adopting the OMOP CDM facilitates efficient, multicentered studies and generation of reproducible evidence. Institutional review board (IRB) review was not required for this data analysis as we de-identified all accessed data elements prior to receipt.^12^ All data queries were completed on February 8, 2023.

### Emergency Department Visits

Following the structure of the OMOP CDM, we identified ED visits from the “visit occurrence” domain using visit concept identifications (IDs) of “9203 – Emergency Room Visit” or “262 – Emergency Room and Inpatient Visit.”^13^

### Opioid-related Events

The ED visits associated with opioid-related events, labelled as “all opioid-related visits,” were identified if they had at least one of the following International Classification of Diseases, 10^th^ ed, Clinical Modification: F11 codes, and T40.0*, T40.1*, T40.2*, T40.3*, T40.4*, T40.6*. Opioid overdose-associated visits were then further classified by types of opioids involved: heroin (T40.1*); prescription opioids (T40.2* or T40.3*); and synthetic opioids other than methadone (T40.4*).^14^

### Outcomes Measures

We summarized and analyzed the outcomes as monthly ED visit rates of opioid-related disorders and overdoses (per 100,000 all-cause ED visits). This was justified by the lower all-cause ED visit rates across the US during the COVID-19 pandemic^15^ and was implemented in previously published studies.^8^ ^,^ ^16^ We also plotted monthly ED visit counts across the UC health centers from 2018–2022 to verify this trend. Actual ED visit counts for opioid-related visits were also masked as per institutional policy.

### Statistical Analysis

We performed interrupted time analysis to estimate the immediate (level) change and change-in-time trend (trend change) with negative binomial regression with robust standard errors (Huber-White sandwich estimator) for over-dispersed outcomes. The model was further adjusted for first-order autoregression using a lag-1 variable (outcome value from the previous month), with all-cause ED visit counts as the offset variable. The model specification is as follows:

$$\log\left(E\left(y_{t}\right)\right)=\beta_{0}+\beta_{1}\cdot covid_{t}+\beta_{2}\cdot time_{t}+\beta_{3}\cdot posttime_{t}+\beta_{4}\cdot lag1_{t}+\log\left(offset_{t}\right)+\varepsilon_{t}$$

•
$y_{t}$ was the count outcome of interest in month t .
•
$covid_{t}$ was a binary dummy variable indicating whether month t was before (January 2018–December 2019, assigned with a value “0”), or during (April 2020–December 2022, assigned with a value “1”) the COVID-19 pandemic. A washout period between January–March 2020 was implemented due to widespread uncertainty regarding the nature of the pandemic during that timeframe.
•
$time_{t}$ was a discrete time variable counting months starting from January 2018, taking the values of 1, 2, 3, etc.
•
$posttime_{t}$ was coded “0” for time points before the COVID-19 pandemic (January 2018–December 2019) and then become a discrete time variable counting months during the COVID-19 pandemic (April 2020–December 2022), taking the values of 1, 2, 3, etc.
•
$lag1_{t}$ was the count outcome of interest in the month prior (ie, $y_{t-1}$ ). This allows control of autocorrelation in a time-series dataset.
•
$offset_{t}$ takes the number of all-cause ED visits in month t . This variable was excluded for the trend analysis of all-cause ED visit counts.
• The key coefficients of interest include the following:
  ◦
$\beta_{1}$ , which represents the immediate (level) change in $\log\left(E\left(y_{t}\right)\right)$ due to the COVID-19 pandemic.
  ◦
$\beta_{2}$ , which represents the monthly change in $\log\left(E\left(y_{t}\right)\right)$ before the COVID-19 pandemic (January 2018–December 2019).
  ◦
$\beta_{2}+\beta_{3}$ , which represents the monthly change in $\log\left(E\left(y_{t}\right)\right)$ during the COVID-19 pandemic (April 2020–December 2022).

Effect sizes for count and rate outcomes are presented as ratios and 95% confidence intervals (CI) via exponentiating the coefficients. Predicted trends overlaying scatterplots were plotted to illustrate the trends in the count and rate outcomes per month. All analyses were tested with α = .05 and completed with Stata v16.1 (StataCorp LLC, College Station, TX).

## RESULTS

### All-Cause ED Visits

**Figure 1.**
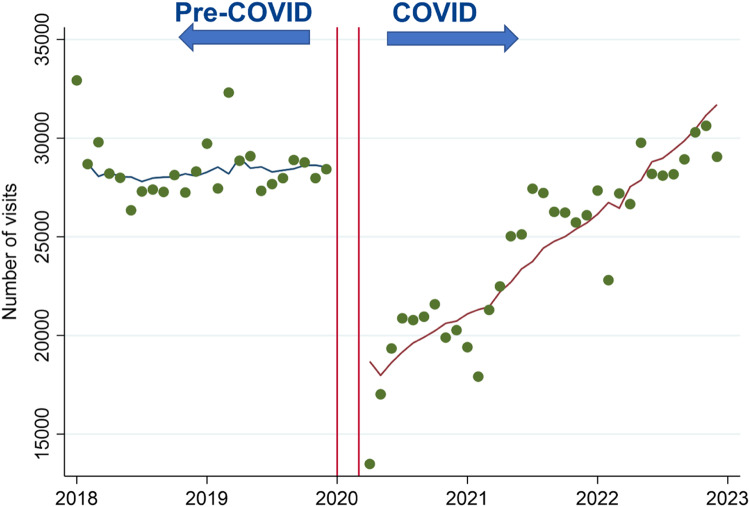
Monthly counts of all-cause emergency (ED) visits from 2018–2022 across six University of California health centers. The period from January 2018–December 2019 was designated as “pre-COVID,” while the period from April 2020–December 2022 was designated as “COVID.” A washout period from January–March 2020 is represented by the red lines in the graph.

At the baseline of December 2019 the total number of ED visits was 28,426 across the six health centers, whereas it fell slightly to 25,121 visits in June 2021. Monthly all-cause ED visit counts remained relatively constant before the COVID-19 pandemic (*P* = 0.30). During the pandemic, there was a sudden decline by 34% compared to pre-pandemic trends (95% CI 15–49%, *P* = 0.002) before increasing by 1.5% per month (95% CI 0.5–2.5%, *P* = 0.002) (Figure 1).

### All Opioid-related Events

**Figure 2.**
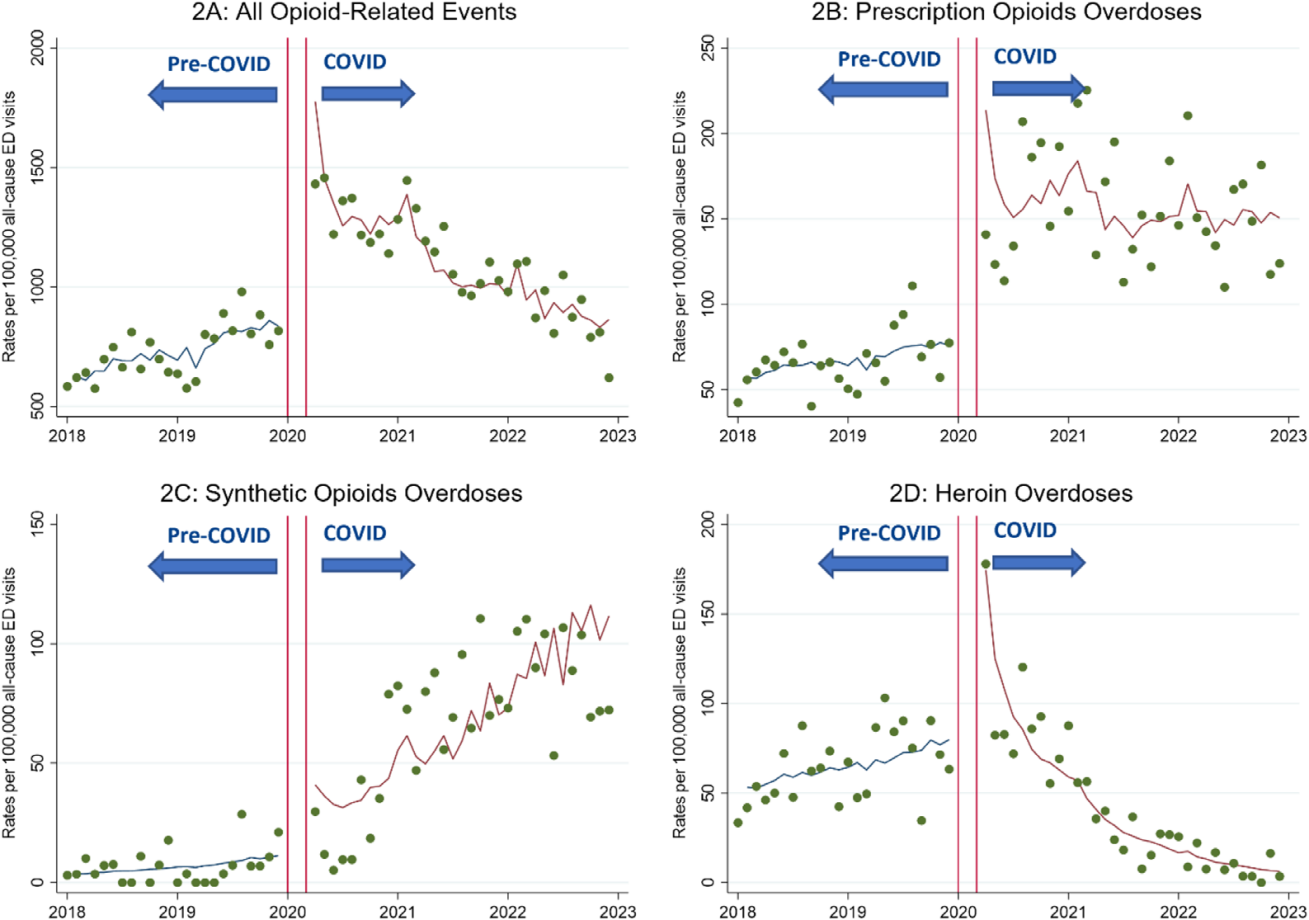
Monthly emergency department (ED) visit rates of opioid-related disorders and overdoses (per 100,000 all-cause ED visits) from 2018–2022 across six University of California Health centers. The period from January 2018– December 2019 was designated as “pre-COVID,” while the period from April 2020–December 2022 was designated as “COVID.” A washout period from January–March 2020 was implemented and is represented by the red lines in the graph.

At the baseline of December 2019 the number of total opioid-related ED visits was 232 across the six health centers, whereas it jumped to 315 visits in June 2021. The ED visit rates of all opioid-related visits increased by 1% per month during the pre-COVID-19 period (95% CI 0.6–2.0%, *P* < 0.001). This was followed by a 15% immediate increase during the pandemic, compared to pre-pandemic levels (95% CI 2–29%, *P* = 0.020), and a statistically non-significant monthly decline by 0.5% (95% CI −0.9 to 0.0%, *P* = 0.06) (Figure 2A).

### Prescription Opioid Overdoses

At the baseline of December 2019 the number of prescription opioid-related ED visits was 22 across the six health centers, whereas it jumped to 49 visits in June 2021. The ED visit rates of prescription opioid overdoses increased by 1% every month before the COVID-19 pandemic (95% CI 0.2–2.0%, *P* = 0.02). During COVID-19, there was an immediate increase in visit rates of 44% (95% CI 10–89%, *P* = 0.008) and a statistically non-significant increase of 1% every month (95% CI −0.2 to 2.0%, *P* = 0.12) (Figure 2B).

### Synthetic Opioid Overdoses

At the baseline of December 2019 the number of synthetic opioid-related ED visits was less than 11 across the six health centers, whereas it jumped slightly to 14 visits in June 2021. Counts less than 11 are masked following the UC Center for Data-driven Insights and Innovation (CDI2) policy on limited research uses using the UCHDW. The ED visit rates of synthetic opioid overdoses increased 5% every month, albeit without statistical significance (95% CI 0.2–11.0%, *P* = 0.06), before the pandemic. During COVID-19, there was a statistically non-significant increase of 69% (95% CI −32 to 316%, *P* = 0.26) compared to pre-pandemic trends, followed by a monthly increase of 4% (95% CI 2–6%, *P* = 0.001) (Figure 2C).

### Heroin Overdoses

At the baseline of December 2019 the number of heroin-related ED visits was 18 across the six health centers, whereas it fell slightly to less than 11 visits in June 2021. As with the counts of synthetic opioid-related ED visits, counts less than 11 are masked per CDI2 policy. Monthly ED visit rates for heroin overdoses increased by 2% monthly during the pre-COVID-19 period (95% CI 0.6–3.0%, *P* = 0.006). Subsequently, the pandemic led to an immediate but statistically non-significant increase by 20% (95% CI −14 to 67%, *P* = 0.28) compared to the pre-COVID-19 period. This was followed by a monthly decline of 8% (95% CI −10 to −7%, *P* < 0.001) (Figure 2D).

## DISCUSSION

The COVID-19 pandemic brought light to the urgent need for multilevel innovative approaches to aid against the opioid epidemic in California. In particular, the pandemic has facilitated the worsening of trends related to prescription and synthetic opioid overdoses across the UC health centers, with little signs of improvement as recent as December 2022. Considering that past research on the same subject was only updated as of 2020 and that our findings were similar to those papers,^5^ ^–^ ^8^ our study may provide the most recent outlook of the current opioid crisis across the US and reflect that more could be done to arrest the problem.

Interestingly, we saw a decreasing trend, albeit non-statistically significant, in ED visit rates related to all types of opioid-related events after an initial spike in April 2020, which contrasted with the trends we saw with opioid overdoses. While the key reasons could not be determined based on the available data, we hypothesize that this decreasing trend could be an artifact of a recovery in numbers of all-cause ED visits. Additionally, this trend possibly reflects a paradigm shift in the seeking of treatment for opioid use disorder from EDs during the pandemic. A key consideration is that opioid-related events comprise not only opioid overdose complications but also opioid withdrawal episodes, and both can be treated at the ED with lifesaving naloxone and buprenorphine, respectively.^17^

The ED also provides these medications for opioid use disorder (MOUD) on discharge as stand-by medications for use as required and connects patients with follow-up OUD management in the community.^18^ ^,^ ^19^ In California, the CA Bridge program has spearheaded the coordinated implementation of low-threshold buprenorphine treatment for opioid withdrawals (an approach that reduces as many barriers as possible regarding access to buprenorphine^20^) at EDs of 52 hospitals, including four UC health centers, since 2018.^21^ Unfortunately, the program reported a steep decline in ED-initiated buprenorphine during the first months of the pandemic.^22^ Our study may provide preliminary evidence that the lower rates of OUD treatment at EDs, especially for withdrawal episodes, have continued into 2021 and 2022. Further research, such as examining rates of administration and prescription of different MOUD at the ED, will be required to confirm this hypothesis.

At the initial stages of the pandemic, Currie et al reported that opioid prescriptions across the US were generally maintained among existing opioid users but decreased briefly for new users.^23^ Another study by Zheng et al reported a decline in opioid prescriptions and prescribers before and during the COVID-19 pandemic period in California.^24^ There were little to no signs of a sudden increase in opioid prescriptions that could explain the higher rates of prescription opioid overdose rates seen in our study. A multisite report studying drug use behaviors during the pandemic found that COVID-19-related stressors, such as job loss, increased housing insecurity, and loneliness, were commonly cited reasons for increasing drug use behaviors.^25^ The same study further reported that synthetic opioids, particularly fentanyl, had saturated the illicit drug market during this time due to its inexpensive nature and high accessibility compared to methamphetamine and heroin.^25^ These trends were further verified in the *California Health Care Almanac* feature on California substance use.^26^ Other than strategizing to reduce the supply of illicit fentanyl and fentanyl-laced pills, improving the mental health of opioid users, and thereby reducing demand for both prescription and synthetic opioids, will also help to mitigate the opioid epidemic more effectively.

Recent policy changes have been made to purposefully bring MOUD closer to patients and prevent deaths from fatal opioid withdrawals and overdoses: 1) the removal of the X-waiver (issued by the Substance Abuse and Mental Health Services Administration and the Drug Enforcement Administration) enabled physicians to prescribe buprenorphine in clinics without administrative barriers and extensive certification^27^ ^,^ ^28^; and 2) the approval by the Food and Drug Administration of Narcan, the first over-the-counter naloxone nasal spray.^29^ However, with the emergence of the fourth wave of the opioid crisis characterized by opioid and stimulant co-use,^30^ the war against the opioid epidemic is not yet won, and effective policy changes will necessitate key changes in current practices and operations.

Varisco et al identified legacy barriers in state-level regulations and wholesaler policy that could limit buprenorphine supplies and dispensing at pharmacies.^31^ Education and the intentional distribution to the community will be required to ensure that Narcan is always available to respond to an opioid overdose event. Finally, there remains evidence of high-risk opioid prescribing and dispensing behaviors associated with a younger and less educated demographic, which may precipitate greater rates of opioid-related cases.^32^ There is still much to do as clinicians, researchers, and community advocates to ensure the effective management of the opioid epidemic, given our available resources.

## STRENGTHS AND LIMITATIONS

One limitation of the study is the use of deidentified data, which prevented us from evaluating individual-level characteristics that are predictive of higher opioid-related admissions or readmissions. Such data will need to be navigated around existing and new regulatory frameworks from multisite IRBs across multiple UC health centers. To our knowledge, this is a hurdle that has yet to be tackled at the UC-wide level, and we suspect that more time will be needed to establish the process. Once established, we will strive to determine sociodemographic and geolocation features that could help with advising the optimal allocation of resources in this war against the opioid epidemic.

Regardless, the strength of this current study lies in the use of real-world ED data to rapidly assess the changes in opioid-related ED trends during the COVID-19 crisis when research data collection had been halted due to lockdown measures. Our study has also demonstrated the feasibility of using this methodology to create a real-time OUD dashboard^33^ to facilitate the timely dissemination of trend data in opioid-related ED visits and admissions in the everchanging, opioid use landscape in California. Additionally, we sought to include community-based programs, such as OUD-based harm reduction organizations, as a beneficial intervention to reduce the number of opioid-related ED visits. However, the UC HDW did not include data that was relevant to these programs, and there is yet to be a robust study displaying their significance.

## CONCLUSION

Our data provides support that opioid-related ED visit rates in California have exceeded pre-pandemic rates and have continued to worsen after 2021. Although the COVID-19 pandemic saw an overall decreasing trend in the number of opioid-related ED visits in all the UC Health centers, the number of visits due to prescription and synthetic opioids-related overdose remains high. More significantly, this increasing trend provides a great public health concern, especially as the US enters the fourth wave of the opioid epidemic, characterized by polysubstance use. Not only must we reduce the supply of illicit opioids, we should also aim to reduce the demand for prescription and synthetic opioids, which is likely the function of worsening mental health during the COVID-19 pandemic. While the COVID-19 pandemic continues to become less notable to the public, the consequential changing landscape of the opioid epidemic remains an uphill battle that calls for multilevel, proactive, innovative, and collaborative approaches across the US.
